# Establishing Shot Type Affects Arousal and Cognitive Load During Transitions Between Novel Interior Locations in Films

**DOI:** 10.3389/fnhum.2019.00003

**Published:** 2019-01-28

**Authors:** Grant Brighter, Nancy Rader

**Affiliations:** ^1^Mind-Body Laboratory, Department of Psychology, Ithaca College, Ithaca, NY, United States; ^2^Cognition Laboratory, Department of Psychology, Ithaca College, Ithaca, NY, United States

**Keywords:** establishing shot, fNIRS, cognitive load, arousal, pupil diameter, continuity editing, functional near-infrared spectroscopy

## Abstract

An “establishing shot” prefaces a scene in a movie with a wide shot of the scene’s location. It is meant to help viewers process a shift to a new location. Establishing shots can depict the actors in the space in which they will be acting, the exterior of a building, or the larger geographic context of the scene. While use of an establishing shot is standard filmmaking practice, some argue that establishing shots are unnecessary. This study sought to investigate how effective four types of establishing shots are at helping viewers process location shifts. Pupil diameter was recorded using a MangoldVision eye tracking system as a measure of arousal and cognitive load. Oxygenation levels in the prefrontal cortex provided an additional measure of cognitive load assessed through functional near infrared spectroscopy (fNIRS). We expected scene transitions to be followed by transient increases in pupil diameter and oxygenation levels, suggesting increased cognitive load and arousal. We predicted that participants should experience less cognitive load and arousal after a transition to a new scene when that scene has been prefaced with an establishing shot and that these effects would be greatest for establishing shots that depict actors. We found that geographic establishing shots produced significantly lower average pupil diameter than all other establishing shot types and the use of no establishing shot. Actors establishing shots elicited significantly lower average pupil diameter values than the use of no establishing shot. Maximum and average oxygenation values for the actors establishing shot condition were significantly higher than for the exterior establishing shot condition. An alpha level of 0.05 was used for all analyses. These results suggest differences between pupil diameter and fNIRS in terms of the psychological phenomenon they measure, and may inform the design of future films.

## Introduction

A standard practice in commercial, narrative filmmaking is the use of establishing shots. An establishing shot is a long^1^, wide shot that prefaces a scene and depicts the location in which the scene will take place. A scene “takes place in one location over a continuous but relatively small amount of time” (Cutting et al., 2012, p. 3); thus, a change in scene is generally linked to a change in location. Pincus and Ascher (2012) succinctly describe an establishing shot as “a long shot that defines the basic space or locale where events will take place” (p. 323).

The present study makes use of three different types of establishing shots. The first is an establishing shot where actors are depicted in a long shot within the space in which they will be acting during the scene. This type of establishing shot, in theory, helps the viewer become accustomed to the fact that they are now occupying a new environment and displays how the characters are positioned at the beginning of the scene. An establishing shot might instead depict the exterior of the building or vehicle where the scene will take place. This could be the front façade of an office building or the outside of a boat or spaceship. In such a shot, the actors often are not depicted, as the following shot usually cuts to an interior space where the action takes place, a space presumably inside the building or vehicle shown in the previous shot. This is an instance that usually does not provide the arrangement of characters, but should, however, cue the viewer to the fact that the location of the narrative has changed. The final type of establishing shot is a long shot of the larger geographic context in which the scene will take place. This type of shot is usually a landscape or cityscape, but it might also take the form of a shot of a planet’s surface. Again, actors are often not depicted in this type of establishing shot.

One thing that all the forms of establishing shot have in common is that they are meant to indicate to the viewer that the location of the action has changed in the film’s ongoing narrative. Cutting and Iricinschi (2015) note that the establishing shot helps a viewer become acclimated to a new location. This “functional” aspect of an establishing shot’s definition makes intuitive sense. One would expect that a long shot of the surroundings before the action of the scene begins in earnest would help the viewer adjust to a shift in location, as it would, in theory, give the viewer a moment to process this shift before focusing on the extension of the narrative delivered in the subsequent scene. Indeed, it is likely that this intuition has helped ensconce the establishing shot as standard filmmaking practice. However, the establishing shot has been argued to be superfluous when it comes to the task of guiding the viewer through a narrative (Bordwell, 2002; Ballester, 2005).

Cutting et al. (2012) found that new units of action tend to be prefaced with wider shots that take in more of the surroundings before moving to closer shots as action unfolds. This study also found that these initial shots were relatively longer in duration than the following shots that made up the scene. The pattern of beginning a scene with a relatively longer shot that is also relatively longer in duration compared to the rest of the shots in the scene was interpreted to be indicative of the usage of establishing shots. In addition, of the various formal aspects of film (features other than place, time, characters, and other narrative content), initial shot scales and initial shot durations were among the variables most consistently linked with the perception of a new event beginning. The research of Cutting et al. suggests that the establishing shot could play an important role in helping the viewer determine that a new event is occurring in the film. However, it is important to note that this research analyzed “subscenes,” which are meant to represent meaningfully distinct events in a narrative (Cutting et al., 2012, p. 3). These can include entrances and exits of characters or shifts of tone. Therefore, the shifts analyzed do not necessarily refer to changes in scene (i.e., changes in location), although such shifts are included in this analysis. So, while Cutting et al.’s results do not specifically represent shifts in locations, they may provide some insight into the importance of the establishing shot for cuing shifts in the narrative, including locational shifts. Indeed, it has been theorized that the establishing shot is kept at a longer duration to give the viewer a chance to take in the novel information being presented, which often takes the form of a shift to a brand new location (Cutting et al., 2012; Cutting and Candan, 2013; Cutting, 2014).

Past studies have found that reading time slows at the beginning and ending of scenes in written stories (Zwaan et al., 1995a,b). These results can be explained by an increased consumption of cognitive resources caused by the transition between scenes (Cutting et al., 2012). Indeed, Zwaan et al. (1995b) have found that general discontinuities in temporal, spatial, and causal elements of a written story lead to increases in reading time, a result that has been interpreted as being reflective of increased difficulty in incorporating new information into preexisting conceptions of the story. It has been postulated that shifts in scenes require heightened executive functioning and place a heavier cognitive load on comprehension (Cutting and Iricinschi, 2015). If narrative shifts such as changes in location in written stories increase processing difficulty, it is possible that such narrative shifts increase processing difficulty in filmic stories as well. Given the seeming influence of the establishing shot on the cognitive distinction between events in films, it is also possible that establishing shots may help reduce the difficulty with which changes in events are processed.

One measure of how difficult it is for a person to process information is known as cognitive load, a term used to refer to the amount of effort required to process information (Cooper, 1990; Zagermann et al., 2016). The current study used functional near infrared spectroscopy (fNIRS) to measure cognitive load via hemodynamic responses in the prefrontal cortex, specifically Brodmann Area 10. Brodmann Area 10 is defined as the medial prefrontal cortex and has been shown to become increasingly activated with increasing cognitive load (Arsalidou et al., 2013).

Functional near infrared spectroscopy has been utilized in a variety of clinical and research settings, and has been validated against other neuroimaging techniques such as PET (León-Carrión and León-Domínguez, 2012). Ayaz et al. (2012) was one of the first studies that established fNIRS as a monitor of cognitive workload. Herff et al. (2014) utilized fNIRS to quantify mental workload activity in the prefrontal cortex during a series of n-back tasks. The fNIRS data showed an increase in oxygenated hemoglobin and a decrease of deoxygenated hemoglobin in the prefrontal cortex as the n-back task increased in difficulty. Workload was evaluated by comparing each n-back trial to one another and to relaxation periods. It was concluded that measuring hemodynamic responses in the prefrontal cortex using fNIRS provides a reliable quantification of mental workload, supporting the findings of Ayaz et al. (2012). Izzetoglu et al. (2004) also found that blood flow in the prefrontal cortex, as measured by fNIRS, correlated with increases in cognitive load. Participants engaged in a naval warfare management simulator task. Blood flow changes as measured by fNIRS were found to be significantly correlated with task difficulty.

In order to be activated, neurons require substances such as oxygen and glucose. Such materials are sent to neurons via blood transfusion through the capillaries. The metabolic needs of neurons during activation results in increased oxygen consumption, local cerebral blood flow, and oxygen delivery (Lloyd-Fox et al., 2010). An increase in blood flow and total hemoglobin to certain regions of the cerebrum is considered to be indicative of increased metabolic demands of the neurons in that region, and therefore, increased activation of those neurons in response to cognitive tasks (Izzetoglu et al., 2004). When hemoglobin is transporting oxygen in the blood, it is referred to as oxygenated hemoglobin; once it releases oxygen to be metabolized, it is referred to as deoxygenated hemoglobin.

Functional near infrared spectroscopy uses infrared light to measure the amount of oxygenated and deoxygenated hemoglobin in areas of the cerebral cortex; in this study we measured activity specifically in the anterior portion of the cerebral cortex. Biological tissue becomes translucent to light under a specific spectrum of 700–900 nm wavelengths, or the near-infrared portion of the light spectrum (León-Carrión and León-Domínguez, 2012). Oxygenated and deoxygenated hemoglobin exhibit different absorption properties when exposed to near-infrared (NIR) light, thus allowing levels of these forms of hemoglobin to be differentiated from one another based on the way NIR light is scattered when it is reflected back from the brain (Lloyd-Fox et al., 2010). A typical hemodynamic response to the activation of cortical neurons in an adult brain takes the form of an increased flow of oxygenated hemoglobin and a relatively smaller decrease in deoxygenated hemoglobin, resulting in an increase in total hemoglobin (Lloyd-Fox et al., 2010). Through measurements of oxygenated and deoxygenated hemoglobin, oxygenation levels in the brain can be deduced. fNIRS has a number of advantages; it is a low-cost system that is quiet, non-invasive, portable, and has relatively good temporal resolution (Ferreri et al., 2014).

To date, there are no known studies investigating the hemodynamic response of adults to film editing techniques using fNIRS. Using EEG, Francuz and Zabielska-Mendyk (2013) found that cuts between unrelated scenes caused higher attention, arousal, and cognitive load than cuts between shots depicting the same scene but from different angles. They note that such heightened mental involvement is often correlated with increased activity in the frontal lobes, particularly the prefrontal cortex. This study did not use unrelated scenes from the same video, however; the clips stitched together were different enough in terms of form and content that the changes between scenes resembled changing channels on a television. Using fMRI imaging and the children’s film *The Red Balloon*, film viewers’ responses to various types of edits have been analyzed (Magliano and Zacks, 2011). Discontinuities in space or time while a character continued an action were found to be associated with decreases in activation of the right lateral superior frontal cortex. However, no changes in brain activation were reported at major scene breaks in the foremost regions of the brain. It is not clear what effect the use of establishing shots had on these results.

Smith (2005) writes that discontinuities, which can include abrupt changes in location or scene, have been shown to cause increases in self-reported arousal as well as physiological responses indicative of greater arousal and attention being directed toward a cut. He also writes that a cut to a new scene or location or other discontinuities of comparable cognitive difficulty can lead to cognitive overload. It is worth noting, however, that there are many forms of discontinuities, and so these results may not necessarily be specific to locational/scenic shifts.

Transitions between unrelated video images have been shown to require greater attention from subjects than shifts between video images that remain within one unaltered narrative scene taking place in one location (Geiger and Reeves, 1993). However, those unrelated clips were from a variety of sources, and therefore transitions between them may not necessarily represent transitions between scenes or locations within one continuous film. Using a similar distinction between related and unrelated images, Lang et al. (1993) found that unrelated cuts consumed more processing capacity.

Pupil diameter is an additional measure that can gauge cognitive load, and has been used for this purpose in a variety of settings (Laeng et al., 2012; Chen and Epps, 2014; Zagermann et al., 2016). Chen and Epps (2014) note that pupil diameter has been found to be a reliable indicator of cognitive load in a wide range of situations. Pupil diameter has also been used as a measure of cognitive load during film viewing (Smith et al., 2006; Swallow et al., 2009; Magliano and Zacks, 2013). Pupil diameter has been found to increase with increasing cognitive demands of a task (Beatty and Lucero-Wagoner, 2000; Zagermann et al., 2016), and around points in films that viewers identified as boundaries between events (Magliano and Zacks, 2013). In addition to measuring cognitive load, pupil diameter can be used as a measure of arousal, with larger pupil sizes indicating greater arousal (Laeng et al., 2012). Cognitive load, arousal, and pupil diameter all seem to be correlated with one another, and their functions may be psychologically integrated. Thus, we intend to infer cognitive load and arousal based on pupil diameter.

We hypothesize that the use of an establishing shot will decrease arousal and cognitive load experienced during the following scene compared to the use of no establishing shot. We anticipate that this effect will be most pronounced for actors establishing shots, moderate for exterior establishing shots, and least pronounced for geographic establishing shots. This is because actors establishing shots are closest to the action that follows in the next scene in terms of scale, while exterior establishing shots are further from the action and geographic establishing shots are the furthest. It is expected that the greater the difference in scale between the establishing shot and the following scene, the greater the logical leap that must be made to connect the two, resulting in more cognitive load and arousal.

## Materials and Methods

### Ethics Statement

This study was carried out in accordance with the recommendations of Policies and Procedures and Guidelines for Investigators, Ithaca College Institutional Review Board. The protocol was approved by the Ithaca College Institutional Review Board. All subjects gave written informed consent in accordance with the Declaration of Helsinki.

### Participants

Thirty-two participants, randomly assigned to each group, were tested. Participants were students from Ithaca College, ranging from 18 to 22 years of age (*M* = 19.75, *SD* = 1.016); 24 of the participants identified as white, and 8 identified as various non-white ethnicities; 25 of the participants were female and 7 were male. Students in psychology classes were recruited mainly by the primary researcher entering psychology classes and promoting the study through an announcement. Psychology professors who offer extra credit for research participation were encouraged to inform their students of the opportunity to partake in this study, along with other studies taking place in the spring semester of 2018. The study was posted on the SONA system. The SONA system is a website that the Ithaca College Psychology department uses to recruit participants for psychological studies run by faculty and students. Students can sign up for studies through SONA, and also receive extra credit by having their participation logged on SONA. Individuals with SONA accounts could see the study displayed under the “studies” tab on SONA.

Students who were taking psychology courses received extra credit for their classes according to the specifications of their professors in exchange for participation. The experimenter reported participation information to the SONA system immediately after participation.

Participants were excluded if they had a severe visual impairment that rendered them incapable of seeing the stimuli presented on a computer screen that was placed about two feet away from them. This did not include people whose impairment had been rectified using contacts, as long as such correctional lenses were being worn at the time the subject participated in the study. This did, however, exclude individuals whose only correctional lenses were glasses, as glasses interfered with eye tracking data collection. Individuals were also precluded from wearing make-up during testing, as make-up could skew the readings of the fNIRS system. These limitations were made clear in the recruitment materials for the study. Through a demographics information form, participants were asked if they needed corrective lenses and, if so, whether they have taken appropriate measures to partake in this study (i.e., wearing contacts) before participating in this study. They were also asked if they were wearing make-up. Prior to beginning the study, participants were asked to sign an informed consent form. No volunteers for this study needed to be excluded.

### Apparatus

#### Functional Near-Infrared Spectroscopy

**Figure 1:**
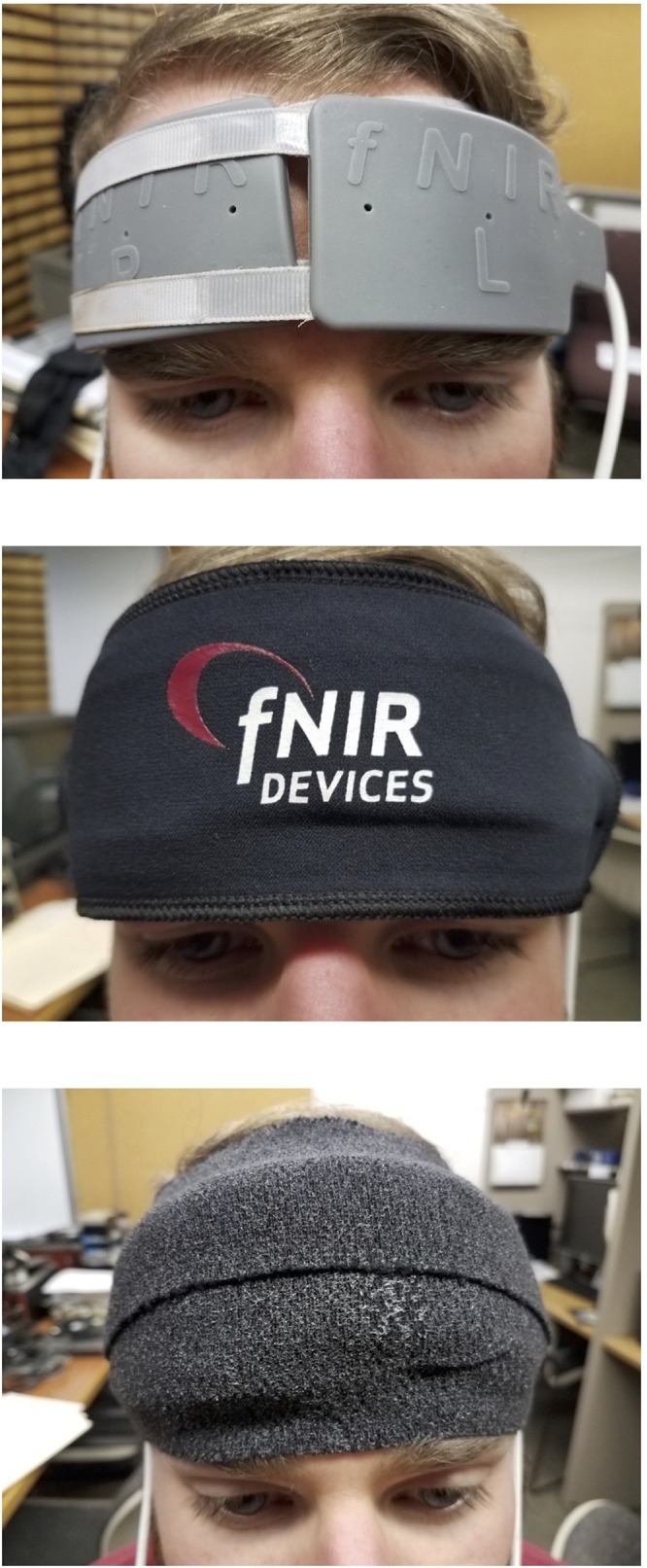
Depiction of sensor placement and wrapping. Each of the two sensors were placed so that the center of the sensor was in line with the center of the pupil and the bottom of the sensor touched the top of the eyebrows of the participant. This can be seen in the top graphic. The sensors were then wrapped with a black band to prevent light from entering, as seen in the middle graphic. Finally, the top and bottom of the band was sealed off from light using gauze strips, as seen in the bottom graphic.

Functional near infrared spectroscopy measurements were made using a continuous wave Biopac system. This system uses sensors placed on the forehead to measure levels of oxygen in the anterior portion of the brain. These sensors are put in place using straps that wrap around the head and are covered with a headband. The bottom of the sensors just touched the tops of the eyebrows, and the center of the sensors were in line with the center of the pupils. The placement of the sensors roughly corresponded to the Fp1 and Fp2 locations in the 10–20 system. To prevent extra light from reaching the sensors, a black band was placed over the sensors, the top and bottom of which was sealed off by wrapping gauze around the participant’s head (see Figure 1). The sensors shine pulses of infrared light into the wearer’s brain. The light interacts with oxygen-carrying proteins in the user’s blood, and the reflected infrared light levels are measured by receptors on the sensor pad. The fNIRS sensor used was a Biopac Systems Inc. RXfNIR-4 Adult 4 Channel Split fNIR Sensor. The fNIRS sensor was split into two headpieces, each with 1 photo-emitter and 2 photo-detectors. Each piece contained two channels. The fNIRS signal measured consisted of the intensity of light reflected back to the sensors at wavelengths of 730 nm and 850 nm. Also, ambient light levels were continuously measured by this system. The fNIRS sensors were cleaned with alcohol and cotton swabs before and after every participant. The sensors were kept clear of hair, as hair could cause artifacts in the data. Participants were provided with headbands, if necessary, to help them keep their hair away from the sensors.

The fNIRS system has an inter-optode distance of 25 mm, and can collect data from up to 12.5 mm beneath the skin (Chance et al., 1993). The fNIRS sensors communicated with a computer through a wireless receiver connected to one of the computer’s USB ports. Levels of light reflected back to the sensors are recorded by COBI studio software and are graphed on the computer monitor during data collection (Ayaz et al., 2011). The fNIRS system had a sampling rate of 4 Hz. fNIRS utilizes the modified Beer-Lambert law to transform optical data into oxy- and deoxy-hemoglobin densities:

$$\Delta \mathrm{OD}\;\;=\;\;\log(I_{\mathrm{rest}}{\mathrm{/I}}_{\mathrm{test}})$$

In this equation, I_rest_ represents reflected light intensity during the baseline condition, and I_test_ represents reflected light intensity while the stimulus is present. ΔOD is change in optical density. By measuring at two different wavelengths within the 700–900 nm range, one above 800 nm and one below, the concentration changes of oxy- hemoglobin and deoxy-hemoglobin can be calculated. The concentrations of oxy- hemoglobin and deoxy-hemoglobin are subsequently used to calculate oxygenation values (Ayaz, 2010).

Oxy and deoxygenated hemoglobin signals were kept between 4000 and 750 mv. To maintain this range, the LED current (brightness) was set between 5 and 20 mA and initial gain (sensitivity) was set to either 1, 5, 10, 15, or 20. The computer used to operate the fNIRS system in this study was a Dell Latitude running Windows 7.

Analysis of the oxygenation levels based on light collected by the sensors were performed using fNIRSoft software. In processing the data, finite impulse response (FIR) and sliding-window motion artifact rejection (SMAR) filters were applied before converting the data to oxygenation values (Ayaz et al., 2010). The FIR filter is a low pass filter with a cut off frequency of 0.1 Hz. To calculate oxygenation values, a baseline level of activity was established using data collected while the viewer watched the last 10 s of a 15 s -long still shot of a vase of flowers at the start of the study. The system then produced oxygenation values that represented differences in activity above or below that baseline. During data collection, the experimenter placed a digital marker in the data right at the end of the shot depicting the vase of flowers. Time bins for analysis accompanying each individual shot were then established based off of the placement of this marker. Once converted to oxygenation values, the data were again subjected to FIR filtering as well as a detrending filter (Ayaz, 2010). New baselines were established using half-second samples at the beginning of each shot, so that oxygenation values reflected differences in activity away from that baseline level. Average and maximum oxygenation values were established for each shot across all optodes. The average and max values across all the shots in the scene were then ascertained to arrive at average and maximum oxygenation for each scene.

#### Pupil Measurement

Eye data were collected using a MangoldVision Eye Tracking System. Through this system, the videos were displayed on a computer monitor. The eye tracking device, which consists of a camera and infrared lights, was positioned underneath the monitor. The infrared light shone on the subject’s face by the lights were registered by the camera in the center of the eye tracking device. The image captured by the camera was used to log the participant’s pupil size throughout the video. Before the video was presented, subjects engaged in a calibration task in which they were asked to look at a red dot that traveled around the computer screen and paused at certain positions. For this study, the dot was programmed to stop at 9 different positions. The participant had to stare at the dot every time it paused in order for the system to calibrate to their unique eyes. If the system was not adequately calibrated, a dialog box appeared notifying the operator and allowing portions of the calibration task to be redone. After the calibration task, the video containing the experimental stimulus began. This is because presentation of this video was controlled by the Mangold system. The screen went blank immediately after the video ended. Data recorded by the Mangold eye tracking system were analyzed using the Mangold Analyzer application. The system was operated by the experimenter using an Inspiron 13 7000 series laptop computer running Windows 10 Pro, which was connected to the eye tracker through a cable. The laptop controlling the Mangold program was separate from the laptop controlling the fNIRS system; thus, two distinct laptops were needed to run the study.

### Stimuli and Procedure

**Figure 2:**
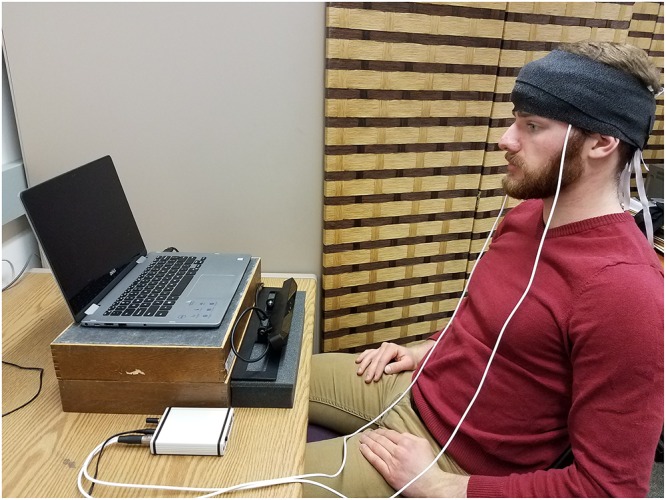
Depiction of experimental set up. The fNIRS sensors were connected to a box (bottom left) that collected the data and wirelessly sent it to the computer running the COBI system. The COBI computer and the experimenter were hidden from the participant by a wooden partition (situated to the right of the individual depicted).

After signing an informed consent form, participants were seated in a chair in front of a laptop computer on which the experimental stimulus was presented. The participants were positioned so that their eyes were approximately 2 feet away from the center of the computer monitor while sitting with their backs pressed against the back of the chair. The computer itself was raised off the desk to a more agreeable viewing height by resting on two wooden stands. The eye tracker was placed on its own foam stand in front of the wooden stands (see Figure 2). The fNIRS sensors were then positioned and wrapped. The overhead lights in the lab were turned off to prevent extraneous light sources from interfering with the readings of the eye tracker and fNIRS system. The eye tracking software was then turned on and the system was calibrated to the participant’s eyes. After the system was calibrated, the experimenter activated the fNIRS system, and then pressed a button that simultaneously instructed the eye tracking software to begin recording and started a video containing the experimental stimulus. The experimenter placed a partition between himself and the participant to minimize distraction (see Figure 2).

**Figure 3:**
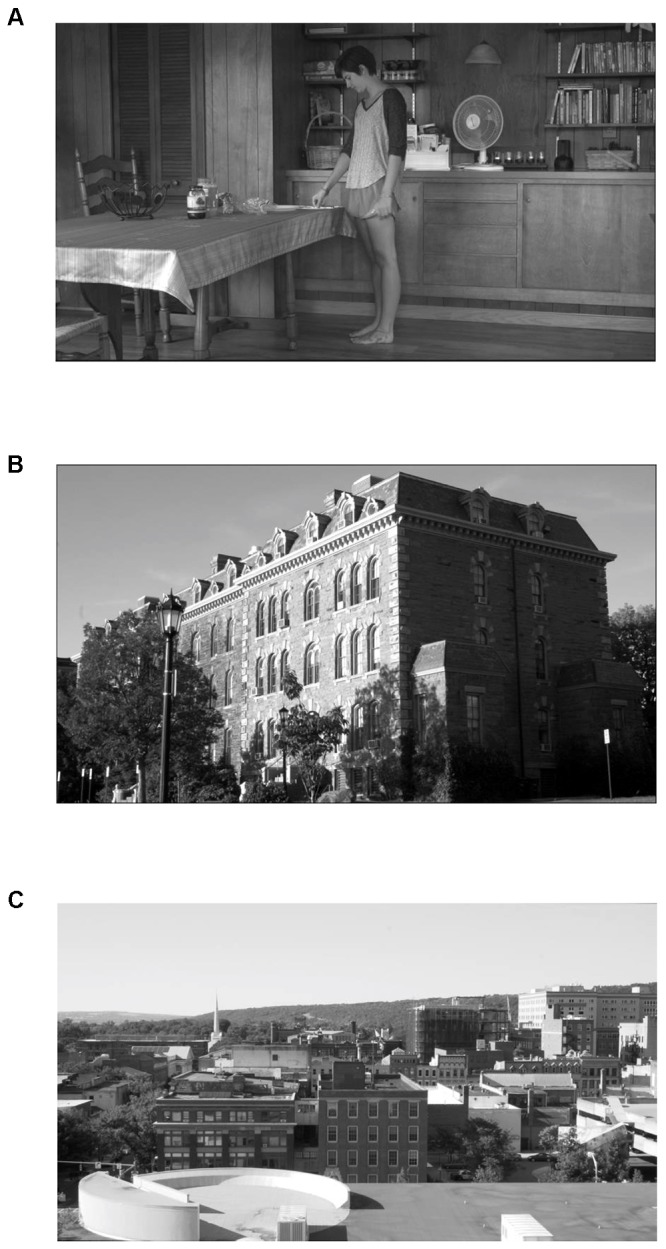
Examples of establishing shot types taken from the experimental stimuli: **(A)** actors establishing shot, **(B)** exterior establishing shot, and **(C)** geographic establishing shot.

The video began with a still image of flowers that stayed on screen for 15 s before the video. The image was taken from an online source of free media. This provided time for the system to calibrate. After 15 s, the fNIRS system began recording data. At the same time, the screen went blank for 2 s, and then the participants were presented with the experimental stimulus, which was a film consisting of five scenes that took place in different locations. The first scene had no establishing shot, but then each of the following scenes started with a different type of establishing shot (see Figure 3):

- An establishing shot where actors were depicted in a long shot within the space in which they were acting during the scene, labeled “Actors.” A long shot was defined as a shot which just barely captured the entire human subject in the frame from head to foot (see Figure 3A).
- An establishing shot that depicted the exterior of the building where the scene took place that was wide enough to capture the entire length and height of the building, labeled “Exterior” (see Figure 3B).
- An establishing shot in which the larger geographic context of the scene was depicted, such as a landscape or cityscape, labeled “Geo” (see Figure 3C).
- No establishing shot (labeled “none”).

Establishing shots were 7.5 s long, except in the “null” condition, in which the establishing shot was 0 s long. The following scenes were 6 shots long. The durations of shots during each scene were: 6, 5.5, 5.25, 5.25, 5.5, and 7 s. The values were based on Cutting et al.’s (2012) approximation of average scene and shot lengths. Also, all the shots in each scene were medium long shots, meaning that the human subject was depicted from head to mid-thigh. Both establishing shots and shots within the scene were framed according to Cutting’s (2014) shot scale definitions. The entire film is 3 min and 33 s long. The aspect ratio of the film (i.e., the ratio of width to height of the image presented onscreen) was 16:9. The film had no audio. All shots were taken from a static frame (no camera movement) and were shot in such a manner that all the visual features within the frame were in reasonably sharp focus. Each scene featured a different actress performing one of the following simple tasks: stretching, making a sandwich, making a bed, drawing on a chalkboard, reading, and drinking from a mug. The actress changed in every scene to disrupt the perception of a continuous narrative between shots. With the exception of the initial shot of the vase of flowers, the entire film was in grayscale.

There were four experimental groups. For every scene after the first scene, each group was presented with a different type of establishing shot. The purpose of this design feature is to prevent a predictable pattern from developing based on the continual use of the same establishing shot, and to emulate the variance in establishing shots seen in real films. The variation in establishing shot type followed the order delineated in Table 1.

**Table 1:** Scene and establishing shot pairings based on group number.

	Scene number			
Group	2	3	4	5
1	None	Geo	Exterior	Actors
2	Actors	None	Geo	Exterior
3	Exterior	Actors	None	Geo
4	Geo	Exterior	Actors	None

*Scene and establishing shot type pairings varied between each film shown to the different experimental groups according to this chart*.

The videos used in this experiment can be viewed by following these links:

• **Group 1: https://youtu.be/LypBfoRfQFE**
• Scene-to-scene content of the video:
• Scene 1: woman pouring water into mug and drinking.
• Scene 2: Woman performing shoulder rolls, stretching shoulders, chest and quadriceps.
• Scene 3: Woman using chalk to connect dots drawn on a chalkboard.
• Scene 4: Woman reading a book.
• Scene 5: Woman making a peanut butter and jelly sandwich.

Establishing shots unique to this video: a long shot of rooftops of downtown Ithaca, a shot of the exterior of a 10-story brick building, a long shot of a woman making peanut butter and jelly.

• **Group 2: https://youtu.be/EukIA3XsRsg**
• Scene-to-scene content of the video: same as Group 1’s video.

Establishing shots unique to this video: long shot of a women performing shoulder rolls, a long shot of rooftops of downtown Ithaca, a shot of the exterior of a house.

• **Group 3: https://youtu.be/Xk3cfcpbN_o**
• Scene-to-scene content of the video: same as Group 1 and 2’s videos.

Establishing shots unique to this video: a shot of the exterior of a 3–4 story building, a long shot of a woman standing in front of a chalkboard, a long shot of rooftops of a neighborhood.

• **Group 4: https://youtu.be/Foa9CUezn5o**
• Scene-to-scene content of the video: same as Group 1, 2, and 3’s videos.

Establishing shots unique to this video: a long shot of the rooftops of downtown Ithaca, a long shot of 3–4 story academic building, a long shot of woman picking up a book.

**Figure 4:**
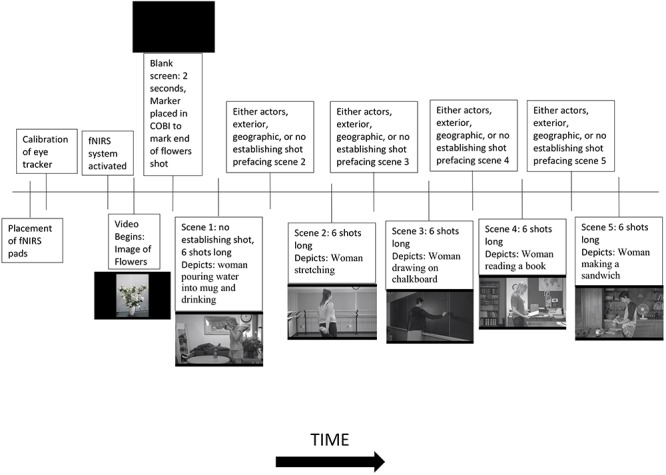
A graphical view of the stimulus presentation procedure.

To see a graphic depiction of the stimulus presentation procedure, please see Figure 4. Once the video stimulus ended, the eye tracking device immediately stopped collecting data. The experimenter then had to manually halt the fNIRS system from recording data.

## Results

Results were analyzed using the statistical package for the social sciences (SPSS).

### Pupil Diameter Analysis

**Figure 5:**
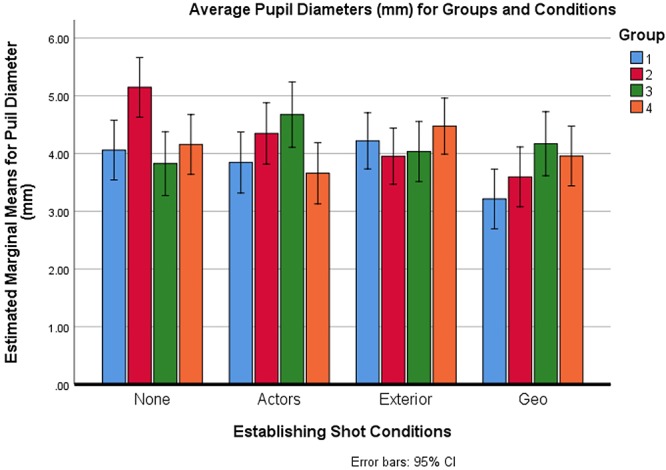
Average pupil diameters for groups and conditions.

Pupil diameters in mm for 31 participants were analyzed using a Mixed Analysis of Variance, with Condition as the within-subject factor and Group as the between-subject factor. One of the original 32 participants was removed from the pupil diameter analysis because the eye tracker was not working for that participant. A significant difference was found between the four establishing shot types *F*(3,81) = 35.096, *p* < 0.0005, η_p_^2^ = 0.565. There was also a group-by-condition interaction *F*(9,81) = 31.683, *p* < 0.0005, η_p_^2^ = 0.779. There was no main effect of group *F*(3,27) = 0.587, *p* > 0.05. A post-hoc Bonferroni test with adjustment for multiple comparisons revealed that geographic establishing shots (*M* = 3.72) were associated with a significantly lower average pupil diameter than no (*M* = 4.32), actors (*M* = 4.12), and external (*M* = 4.17) establishing shots; all p values were less than *p* = 0.0005, two-tailed. In addition, the no establishing shot condition was significantly higher than the actors establishing shot condition (*p* = 0.015) (see Figure 5).

### Maximum Oxygenation Analysis

**Figure 6:**
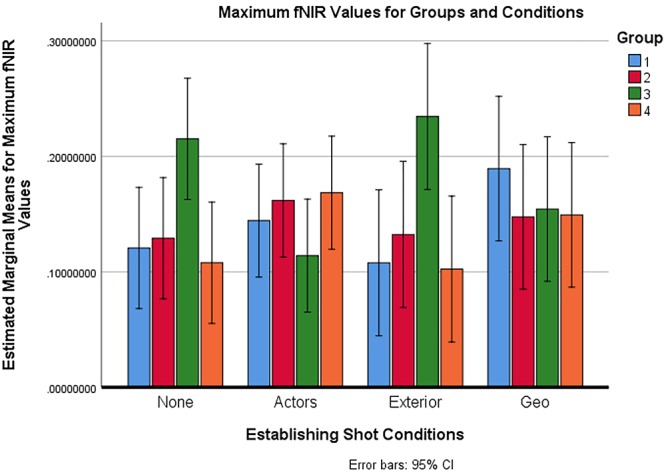
Maximum fNIRS values across all optodes for groups and conditions.

Maximum fNIRS values were analyzed for 32 participants using a Mixed Analysis of Variance as above. The dependent measure was the maximum oxygenation value across all the shots in a scene and across all optodes, which was the maximum change in oxygenation away from baseline. Values were calculated based off of a local baseline established at the first half-second of every shot. There was no significant difference found in maximum oxygenation value based on the four establishing shot types *F*(3,84) = 0.403, *p* > 0.05. However, there was a significant condition by group interaction: *F*(9,84) = 2.946, *p* = 0.004, η_p_^2^ = 0.240. There was no main effect of group *F*(3,28) = 1.373, *p* > 0.05. Based on an examination of the data, a follow-up mixed ANOVA was performed without Group 3. In this follow-up analysis, it was found that the groups did not differ *F*(2,21) = 0.169, *p* = 0.845, η_p_^2^ = 0.016. Also, no group-by-condition interaction was found *F*(6,63) = 0.533, *p* = 0.781, η_p_^2^ = 0.048. There was, however, for Groups 1, 2, and 4, a main effect of condition *F*(3,63) = 3.197, *p* = 0.029, η_p_^2^ = 0.132. A post-hoc Bonferroni test with adjustment for multiple comparisons showed that the values associated with the actor establishing shot condition were significantly higher than the exterior establishing shot condition (*p* = 0.037). For means with error bars, see Figure 6.

### Average Oxygenation Analysis

**Figure 7:**
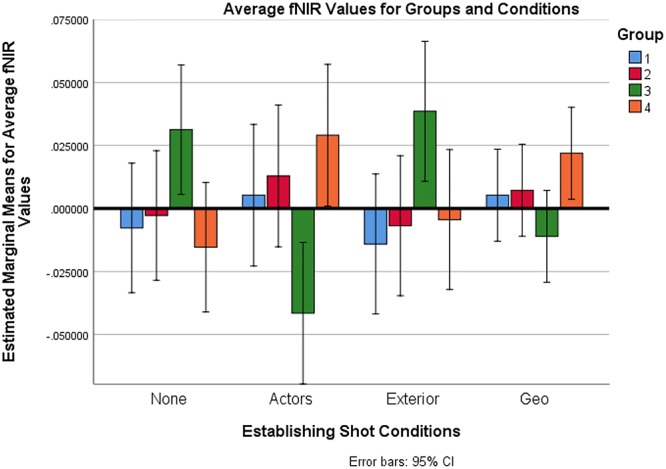
Average fNIRS values across all optodes for groups and conditions.

Average fNIRS values across a scene were analyzed for 32 participants using a Mixed Analysis of Variance as described above. Values were calculated based off of a local baseline established at the first half-second of every shot. The dependent measure was the average oxygenation value across all the shots in a scene and across all optodes, which was the average change in oxygenation away from baseline. There was no significant difference found in average oxygenation value for the four establishing shot types *F*(3,84) = 0.1, *p* = 0.960. However, there was a significant condition by group interaction: *F*(9,84) = 3.767, *p* = 0.001, η_p_^2^ = 0.288. There was no main effect of group *F*(3,28) = 1.069, *p* = 0.378. As with the maximum values, a follow-up mixed ANOVA was performed without Group 3. In this follow-up analysis, it was found that the groups did not differ *F*(2,21) = 2.064, *p* = 0.152. Also, no group-by-condition interaction was found *F*(6,63) = 0.489, *p* = 0.815. There was, however, a main effect of condition *F*(3,63) = 4.007, *p* = 0.011, η_p_^2^ = 0.160. A post-hoc Bonferroni test with adjustment for multiple comparisons showed that the actor establishing shot condition was significantly higher than the exterior establishing shot condition (*p* = 0.016). Means with error bars are presented in Figure 7.

## Discussion

The goal of this study was to determine the degree to which the use of the various forms of the establishing shot help a viewer process shifts to new interior locations within a film by measuring oxygenation in the prefrontal cortex and pupil diameter. It was hypothesized that the use of actors establishing shot would cause the lowest levels of oxygenation and pupil diameter, followed by exterior, geographic, and finally no establishing shot. However, for average pupil diameter, geographic establishing shots caused significantly lower average pupil diameter than all other establishing shot types and no establishing shot. In addition, the actors establishing shot caused significantly lower average pupil diameters than no establishing shot. Initially, no main effect of establishing shot type was found for maximum or average oxygenation values in a scene. However, there was a group-by-condition interaction for both of these measures caused by the values from group 3. When group 3 was removed from the analysis, however, both the maximum and average oxygenation values for the actor establishing shot condition were significantly higher than for the exterior establishing shot condition.

The average oxygenation values corroborate the findings of the maximum oxygenation values. Both average and maximum oxygenation values associated with exterior establishing shots were significantly lower than those associated with actors establishing shots. Thus, it appears that exterior establishing shots are better at reducing cognitive load during scene transitions than actors establishing shots. In contrast, there is no difference between exterior establishing shots and the use of no establishing shot in terms of arousal. In terms of workload, none of the establishing shot types were found to differ from no establishing shot. The addition of the establishing shot does nothing to help make the film transition easier to process. Thus, the advantage of the use of the establishing shot is through its ability to reduce arousal, not its ability to reduce the recruitment of cognitive resources needed to process a scene transition.

The differences between the results of the pupil diameter and fNIRS data may indicate the difference in the way establishing shots affect arousal versus cognitive load. Pupil diameter is thought to measure arousal and cognitive load, while fNIRS only measures cognitive load. It was found that the data diverged across the two measures: actor establishing shots caused greater oxygenation than exterior establishing shots, but no such pattern was found with pupil diameter. Instead, pupil diameter was minimized with geographic establishing shots, and the use of no establishing shot caused higher average pupil diameter than the use of actors establishing shots. Because the measures disagree, it would seem that scene changes prefaced by exterior actor establishing shots are easier to process than actor establishing shots, but that actors establishing shots elicited less arousal than no establishing shots and that geographic establishing shots cause less arousal than all other establishing shot conditions.

The divergence of arousal and cognitive load might be explicable through neuroanatomy. Perhaps arousal and cognitive load are mediated by two completely different brain systems: arousal by the limbic system, and cognitive load by cortical areas. This might explain why the eye tracking data and fNIRS data do not coincide.

There was a group-by-condition interaction in the pupil diameter data, and in the fNIRS data when group 3 was included. This could indicate that the order in which a particular establishing shot was presented affected the results. It is also possible that the specific pairing of the establishing shot and scene had an effect as well. In addition, the specific shots used for each type of establishing shot differed across groups. For instance, a different building is featured in the exterior establishing shot in each group. This was because the importance of having an establishing shot that could plausibly be paired with the following scene’s location was prioritized over having consistency in the image presented across groups. In the case of group 3, the particular images selected as establishing shots may have created especially jarring scene transitions, which may explain its influence on the fNIRS results. Pupil diameter values may have also been influenced by differing levels of luminance in different establishing shots or scenes, causing the group-by-condition interaction.

Geographic establishing shots as well as actors establishing shots were both found to cause less arousal than using no establishing shot, seeming to indicate that using these types of establishing shots can indeed create smoother transitions between scenes. This would indicate that filmmakers’ and film theorist’s intuitions have been partially validated; for specific types of establishing shot, the use of the establishing shot as a means of smoothing scene transitions has been upheld. Specifically, geographic establishing shots seem to be best at performing their intended function, and may be worth using more often in future films. However, the establishing shot’s effect may be because the sample in this study is accustomed to scenes in novel locations being prefaced with actors and geographic establishing shots, and so the absence of these types of shots during scene transitions seems jarring. Indeed, Schwan and Ildirar (2010) suggest that comprehension of the establishing shot technique is not naturally intuited by first-time film viewers, but is conditioned through repeated exposure to film. It is also possible that, despite being jarring, the transitions using no establishing shot may not have needed enough cognitive resources during their processing to be associated with any detectable increase in cognitive load.

From these results, it is concluded that having an actors establishing shot or geographic establishing shot can smooth transitions between scenes by decreasing arousal but not by decreasing cognitive workload. Exterior establishing shots have no effect on arousal or cognitive load compared to forgoing the establishing shot.

This study had several important limitations. The first was sample size; each group only had seven to eight participants, which limits the amount of confidence that one can invest in the significant results found. Further, since the experimental stimulus was not designed to have a coherent narrative, it does not wholly resemble a commercial, narrative film. Thus, future studies may want to explore the effect of the establishing shot while participants are watching a widely distributed feature-length commercial film as a means of generalizing the results found in this study. Future studies may also want to investigate the different areas of the brain responsible for arousal and cognitive load.

## Data Availability Statement

The raw data supporting the conclusions of this manuscript will be made available by the authors, without undue reservation, to any qualified researcher.

## Author Contributions

GB was responsible for reviewing existing literature, developing the experimental design, creating the experimental stimulus, and conducting the experiments. NR advised GB throughout these steps. GB and NR were responsible for preparing the data for analysis and interpreting the results. NR ran statistical tests on the data. GB wrote the manuscript that was submitted for publication. NR edited the manuscript before it was submitted.

## Conflict of Interest Statement

The authors declare that the research was conducted in the absence of any commercial or financial relationships that could be construed as a potential conflict of interest.

## Footnotes

1 In accordance with filmmaking conventions, a shot is regarded as long when the camera is a relatively long distance away from the subject and when a relatively low focal length is used, resulting in a substantial swath of the surroundings around the subject being captured in the shot. Thus, a *long shot* refers to shot scale/distance, whereas the term *long take* is a temporal reference (specifically, it describes a shot of relatively long duration).
